# Novel Design of the Compound Sleeve and Stem Prosthesis for Treatment of Proximal Femur Bone Defects Based on Topology Optimization

**DOI:** 10.3389/fbioe.2022.938337

**Published:** 2022-06-24

**Authors:** Haowen Xue, Haotian Bai, Rongqi Zhou, Jincheng Wang, Bin Zhou, Xiaonan Wang, Wenbin Luo, Xin Zhao

**Affiliations:** ^1^ Department of Orthopedics, the Second Hospital of Jilin University, Changchun, China; ^2^ The Second Clinical Medical College, Jilin University, Changchun, China

**Keywords:** finite element analysis, topology optimization, porous structure, bone defect, prosthesis design, proximal femur

## Abstract

The loosening of traditional prosthetics is among the leading causes of surgical failure of proximal femoral bone defects. A novel compound sleeve and stem prosthesis was designed using an optimization methodology that combined an octet-truss porous structure with density-based topology optimization to improve stability, promote bone ingrowth, and enhance biomechanical properties. Biomechanical changes were assessed using finite element analysis. The distribution of stress, the strain energy density, and the relative micromotion in the optimized group were considered. The optimized sleeve prosthesis achieved a 31.5% weight reduction. The maximum stresses in the optimized group were observed to decrease by 30.33 and 4.74% at the back sleeve and neck part of stem prosthesis, with a 29.52% increase in the femur, respectively. The average stress in most selected regions in the optimized group was significantly greater than that in the original group (*p* < 0.05). The maximum relative micromotion decreased by 15.18% (from 63.9 to 54.2 μm) in the optimized group. The novel designed compound sleeve and stem prosthesis could effectively improve the biomechanical performance of next-generation prosthetics and provide a microenvironment for bone ingrowth. The presented method could serve as a model for clinical practice and a platform for future orthopedic surgery applications.

## Introduction

Proximal femoral bone defects often occur in patients with bone tumors, comminuted intertrochanteric fractures, and a history of revision hip arthroplasty (Gocer et al., 2016; De Martino et al., 2019; Toepfer et al., 2021). Due to substantial bone loss and altered anatomical features, the treatments are often challenging to implement. Several types of prostheses have been used in recent surgeries, including extension biotype stems and tumor prostheses for tight press-fit fixation through the distal femoral isthmus (Hasegawa et al., 2021). Porous structural femoral stems have been developed as biotype stems to achieve biological fixation by bone ingrowth, as proved in clinical and experimental practice (Wallace et al., 2020). However, long-term follow-up has revealed that current-generation prostheses with isthmus fixation appear to fail, and their loosening is often characterized by bone loss and compromises revision and anchorage of further implants (Perry et al., 2016). Furthermore, preserving bone stock is especially crucial in patients with bone defects. As a result, it is necessary to create a novel prosthesis to improve the stability of prostheses and avoid bone resorption.

In current total hip arthroplasty (THA), significant peri-implant bone resorption and aseptic loosening can occur as a result of several factors, including stress shielding and wear failure (Wang, et al., 2018; Bartolomeu et al., 2019a). The primary reason for bone resorption and prosthesis loosening secondary to stress shielding is that the orthopedic implants that are stiffer than bone limit the transfer of a load to bones (Moyen et al., 1978; Zhang et al., 2020; Liu et al., 2021). According to Wolff’s law, homeostatic mechanisms shift toward a catabolic state if the load on a bone is reduced (Frost, 1994). In order to ameliorate the mismatch of stiffness between the implant and the adjacent host bone, most relevant research work has focused on developing new porous metal materials, surface treatments, and prosthesis geometry designs (Wang et al., 2018; Bai et al., 2019). However, conventional THA stems are prone to causing stress shielding with proximal unloading and more distal load transfer (Yan et al., 2020; Hamilton, 2021). Short-stem prostheses have received increased attention in recent years owing to their bone-protective properties, which provide favorable conditions for revision and biomechanical advantages to reduce stress shielding (Bieger et al., 2013; Boese et al., 2016). However, using a short-stem prosthesis to treat proximal femoral bone defects is still under debate. Hence, a sleeve prosthesis was designed based on the principle of short-stem proximal fixation.

Topology optimization (TO) is a structural design approach that provides the optimal material distribution to reduce higher strength-to-weight ratios by eliminating material from the point of lowest stress while retaining high-stress regions (Zhang et al., 2020). Meanwhile, graded porous structures may be added to the optimized region of implants to provide a bone growth microenvironment and control surface modifications on the premise of providing biomechanical properties (Rahimizadeh et al., 2018; Bartolomeu et al., 2019a; Bai et al., 2019). Furthermore, several studies have concluded that less stiff materials and rougher surfaces generate better initial micromotions, which can be used to predict the process of osseointegration with adequate long-term stability (Pilliar et al., 1986; Alkhatib et al., 2019). To our knowledge, no relevant study has yet designed a proximal femoral sleeve prosthesis based on the TO technique. In addition, to further assess design rationality, finite element analysis (FEA) can be used to demonstrate differences in biomechanical conditions during the pre-clinical evaluation.

Therefore, this study proposed a novel TO prosthesis design for addressing bone defects in the proximal femur. According to a rational analysis of prosthesis and bone biomechanical properties, the new product could optimize the design of a sleeve prosthesis, secure the strength, and provide a biomechanical environment for bone ingrowth and long-term stability.

## Materials and Methods

### Geometry and Meshing

**FIGURE 1:**
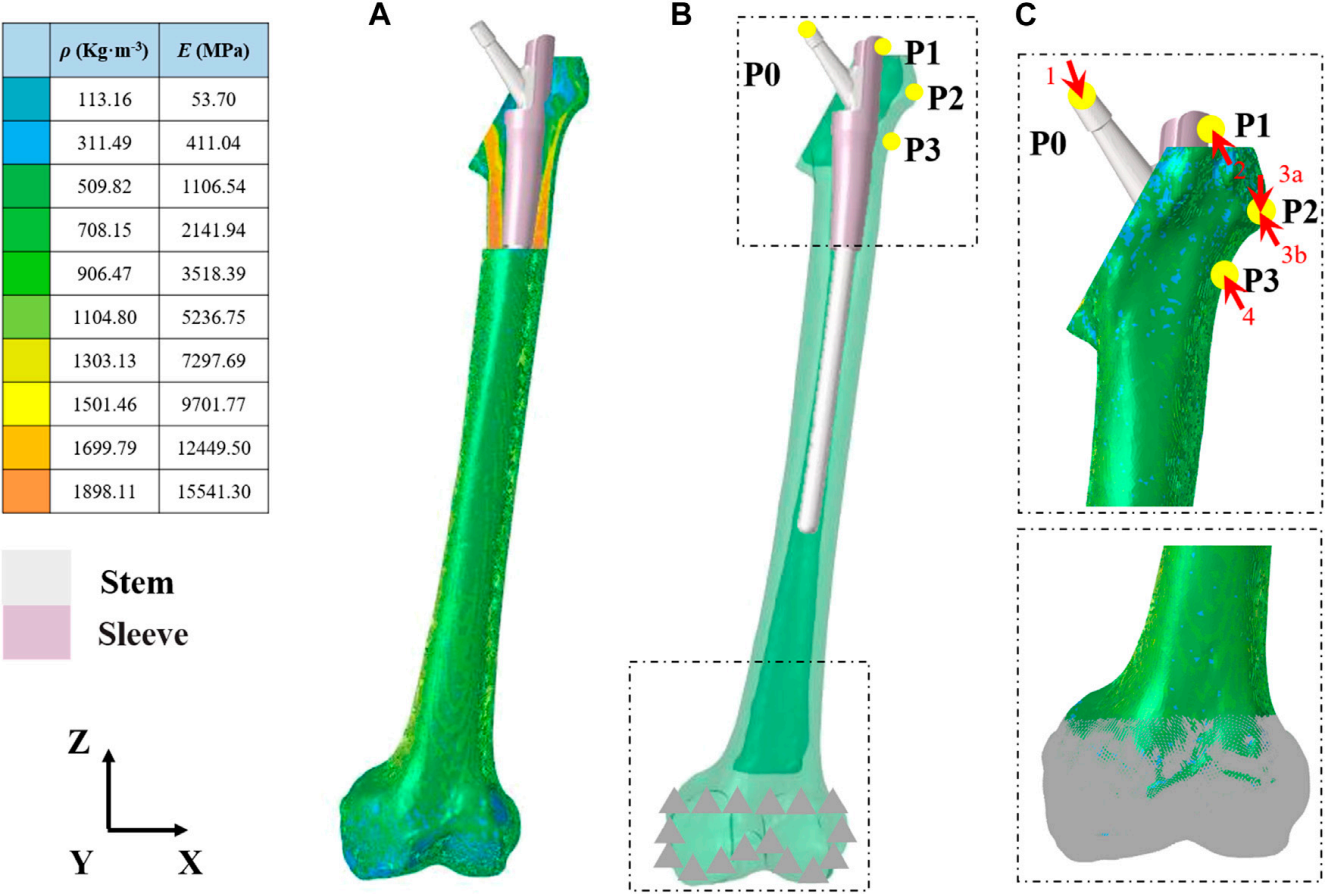
Finite element model of the femur and prosthesis **(A)** Material properties of the inhomogeneous femur **(B)** The entire assembled model of the femur and prosthesis **(C)** Load and boundary conditions. Arrowheads and triangles indicate the loads and constraint, respectively. P0–P4 indicate the point of muscle attachment.

Computationally designed novel implants were constructed using SolidWorks version 2021 (Dassault Systèmes, Vélizy-Villacoublay, France). The implant included an intramedullary needle–type stem prosthesis (length, 225 mm; diameter, 10 mm) and a proximal femoral sleeve (inner height, 65 mm; outer height, 100 mm; inner diameter, 10 mm; outer diameter, 15 mm) (Figure 1). A series of CT images from the imaging database of the Second Hospital of Jilin University were included in the present study. After excluding femur deformity, osteoporosis and any other factors that may change the normal femur anatomies such as fracture, infection, and tumor, a healthy 66-year-old male volunteer (weight, 75 kg) was selected. The femur 3-dimensional model from this volunteer was created via computed tomography (CT) imaging using the iCT 256 C T scanner (Philips, Eindhoven, Netherlands) with a slice thickness of 0.602 mm at 156 mA and 120 kVp. The medical image processing software program Mimics (version 19.0; Materialise, Leuven, Belgium) was applied to reconstruct the 3-dimensional model using the CT data and then convert this model into STL format. Magics version 21.0 (Materialise, Leuven, Belgium) was used to create an Evans type III intertrochanteric fracture bone defect to mimic a proximal femur bone defect (Evans, 1949). The femur was first resected at a 60-degree inclination horizontally from the lesser trochanter. Then, another horizontal resection was performed to generate the bone defect through the midpoint of the greater trochanter. For simplification purposes, the effects of the ligaments were neglected in the models. This study was approved by the ethics committee of the Second Hospital of Jilin University, and informed consent was obtained from all volunteers.

HyperMesh version 2020 (Altair Engineering, Troy, MI, United States ) was used to generate triangular meshes and convert them into 4-node linear tetrahedron elements (C3D4). A sensitivity analysis of mesh quality was carried out until a change of <5% in the maximum principal stress was obtained by mesh refinement. Finally, an average mesh size of 1 mm was set onto the designed components.

### Material Properties

The femur was defined as inhomogeneous with assigned material properties in Mimics for bone density (*ρ*) (Kg/m^3^), elastic modulus (*E*) (MPa), and Poisson’s ratio (*ν*) according to the gray values of the CT scan (Figure 1A and Table 1). According to the previous literature, Poisson’s ratio was set to 0.30, and the bone density and elastic modulus of the femur were determined based on the following formulae (Rho et al., 1995):

$$\text{ρ}\left(Kg/m^{3}\right)=131+1.067\times GV\left(HU\right)\;\;\;\;\;\;\;\;\;\;$$ (1)

$$E\left(MPa\right)=0.004\times \rho^{2.01}\left(Kg/m^{3}\right)\;\;\;\;\;\;\;\;\;\;\;\;\;\;\;\;$$ (2)

**TABLE 1:** Material properties of components.

Component	Material	Elastic Modulus (MPa)	Poisson’s Ratio
Bone	Non-homogeneous	Non-homogeneous	0.30
Stem prosthesis	Ti_6_Al_4_V	110,000	0.34
Sleeve prosthesis	Ti_6_Al_4_V	110,000	0.34
200-μm porous part	Ti_6_Al_4_V	35,849.6	0.34
400-μm porous part	Ti_6_Al_4_V	28,766.8	0.34

The intramedullary needle–type stem prosthesis and proximal femoral sleeve were linear, homogeneous, and isotropic (Ti_6_Al_4_V, *E* = 110 GPa, *ν* = 0.34) (Bartolomeu et al., 2019b). The elastic modulus of the graded porous structures was obtained from a mathematical relationship between the design open-cell ( x ) (μm) and wall size (y) (μm), as follows (Bartolomeu et al., 2019b):

$$E\left(MPa\right)=39.7205-0.1217x+0.4415y+9.4232\times 10^{-5}x^{2}-5.1581\times 10^{-4}xy-8.6838\times 10^{-5}y^{2}\;$$ (3)

An octet-truss lattice architecture with a porosity of 70% was selected to design the graded porous structures ( $x_{1}=200\;\text{μm},\;y_{1}=50\;\text{μm},\;E_{1}=35849.6\;MPa;\;x_{2}=400\;\text{μm},\;y_{2}=100\;\text{μm},E_{2}=28766.8\;MPa$ ) under the constraints of bone growth and electron beam melting (Bartolomeu et al., 2019a). Related material properties are presented in Figure 1A and Table 1.

### Load and Boundary Conditions

The loading conditions of this study adopted the results from Heller et al. (2005), who took the effects of joint and muscle strength into account under a normal walking gait. Corresponding loads were applied to the proximal femur and the prosthesis and presented in Figures 1B,C and Table 2. The friction response between Ti_6_Al_4_V and its porous surface against bone was applied in the research by Bartolomeu et al., 2019a. Sticky contact was set for the interface sleeve and intramedullary needle–type stem prosthesis. The contact surfaces were considered to exhibit non-linear contact conditions, and the friction coefficient is shown in Table 3. The nodes at the contact surface between different components were set in the common spatial location to create closed gaps. The nodes on the superior surface of the distal femur were fully constrained for all degrees of freedom (DOFs).

**TABLE 2:** The joint and muscle forces under walking conditions.

Force	Acts at Point	X	y	z
Hip contact (1)	P0	370.6	225.2	−1,572.3
Abductor (2)	P1	−397.0	−29.5	593.4
Tensor fascia latae, proximal part (3a)	P2	−49.4	−79.6	90.6
Tensor fascia latae, distal part (3 b)		3.4	4.8	−130.3
Vastus lateralis (4)	P3	6.1	−126.9	−637.3

**Note:** Loading conditions refer to Figure 1C.

**TABLE 3:** Friction types between components.

Contact Surface A	Contact Surface B	Friction Type
Sleeve	Stem	Stick
Sleeve	Bone	*μ*_1_ = 0.713, *μ*_2_ = 0.326
200-μm porous part	Bone	*μ*_1_ = 0.667, *μ*_2_ = 0.431
400-μm porous part	Bone	*μ*_1_ = 0.660, *μ*_2_ = 0.459

**Note:** μ_1_ is defined as the static friction coefficient and μ_2_ is defined as the kinetic friction coefficient.

### TO

An optimal design was determined from the TO process by modifying the material’s distribution. The central regions providing strength elements from the analysis would be preserved, and the optimized regions would be modified as porous structures to increase friction. The optimum purpose was that the sleeve prosthesis would be effectively designed to strengthen stability, reduce relative micromotion, and provide the biomechanical environment for bone ingrowth. Moreover, the newly designed prosthesis would maintain the original shape. Hence, the minimum compliance of the TO subject to a volume fraction constraint was utilized under the loads and boundary conditions mentioned above.

The Optimization Equation Is as Follows

Objective Function: Minimize (Uc)

**FIGURE 2:**
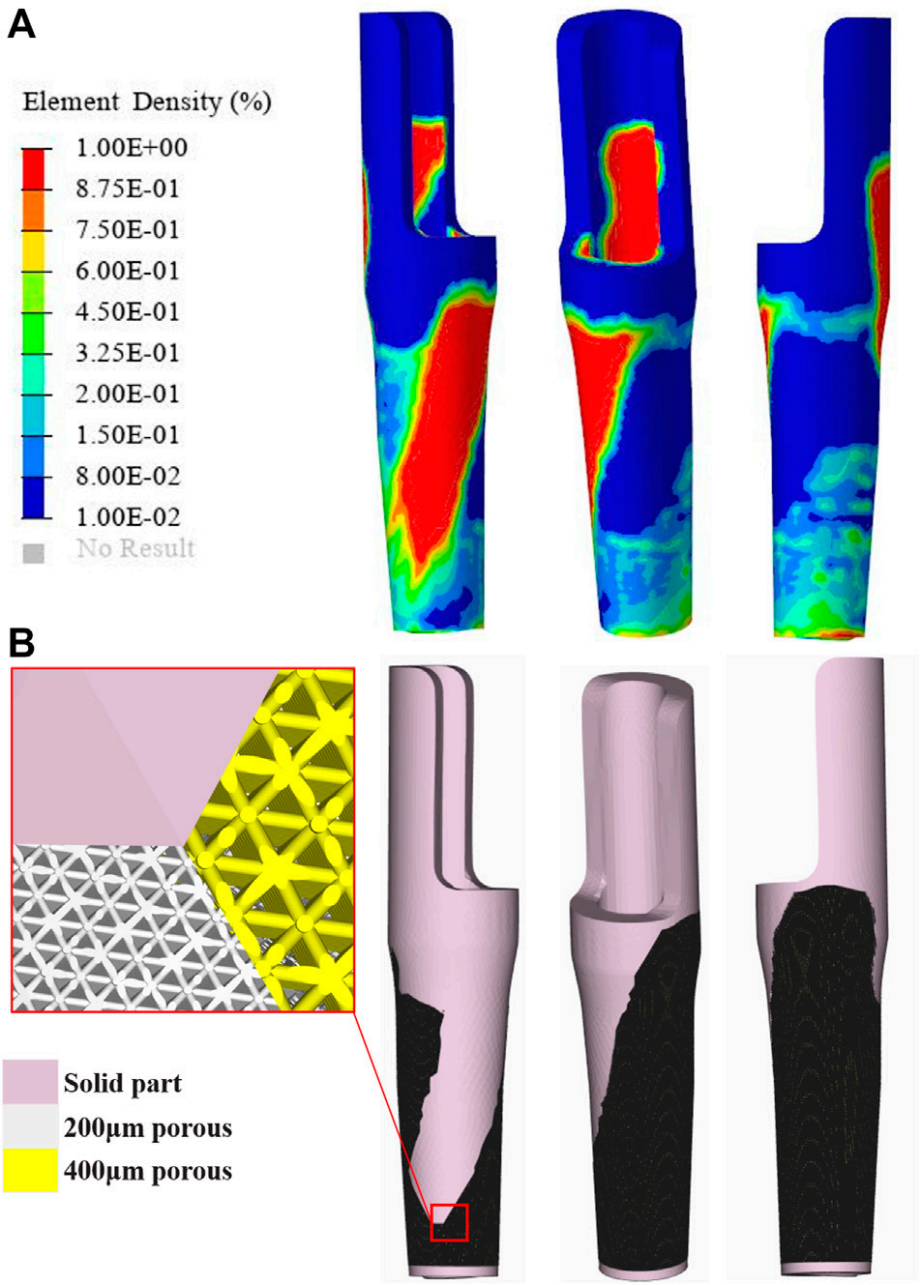
Topological optimization process and the porous structure sleeve prosthesis **(A)** The result of element density distribution **(B)** Optimized prosthesis and its porous structure.

Constraint: 0 < ηi < 1 (i = 1, 2, 3 … n)

$$V\leq V_{0}-V\text{*},$$

$$V=\underset{i}{\sum }\eta_{i}V_{i},$$

$$E_{i}=E\left(\eta_{i}\right),$$

and

$$\left\{\sigma_{i}\right\}=\left[E_{i}\right]\left\{\varepsilon_{i}\right\},$$

where Uc is the compliance, ηi represents the internal pseudo-densities assigned to each finite element (i) in the optimization equation, V is the computed volume, V0 is the original volume, V* represents the amount of volume to be removed, Vi is the volume of element i, Ei is the elasticity tensor for each element, E represents the elasticity tensor, σi is the stress vector of element i, and εi represents the strain vector of the element. η, as the density index, ranges from 0 to 1; an η value close to 0 indicates the material to be removed, and an η value close to 1 indicates the material to be retained. The program is set to reduce the volume by ≤ 20% and to iterate up to 30 times. The convergence tolerance was 0.0001. In the TO postprocessing software Hyperview (Altair Engineering, Troy, MI, United States ), the optimized region was divided into 2 parts according to the volume fraction chosen for 40–60% and 60–100%, respectively. To prevent porous structure collapse in the area of contact with the intramedullary needle–type stem prosthesis and to assure sufficient strength, the top and bottom contact parts of the sleeve prosthesis were preserved. Then, the optimized parts were imported into the Magics software program to design the internal architecture and assemble optimized parts with the preserved part. The optimized parts were designed as a 70% porosity octet-truss lattice structure, which was a good choice, being combined with stable mechanical properties that promote bone formation (Arabnejad et al., 2016; Feng et al., 2021). Depending on the different choices of volume fraction, a pore size of 200 μm or 400 μm was filled to the region of 40–60% or 60–100% to provide different amounts of roughness, promoting proper bone ingrowth (El Elmi et al., 2020). Then, the optimized parts and the preserved parts were fixed together (Figure 2).

### FEM

**FIGURE 3:**
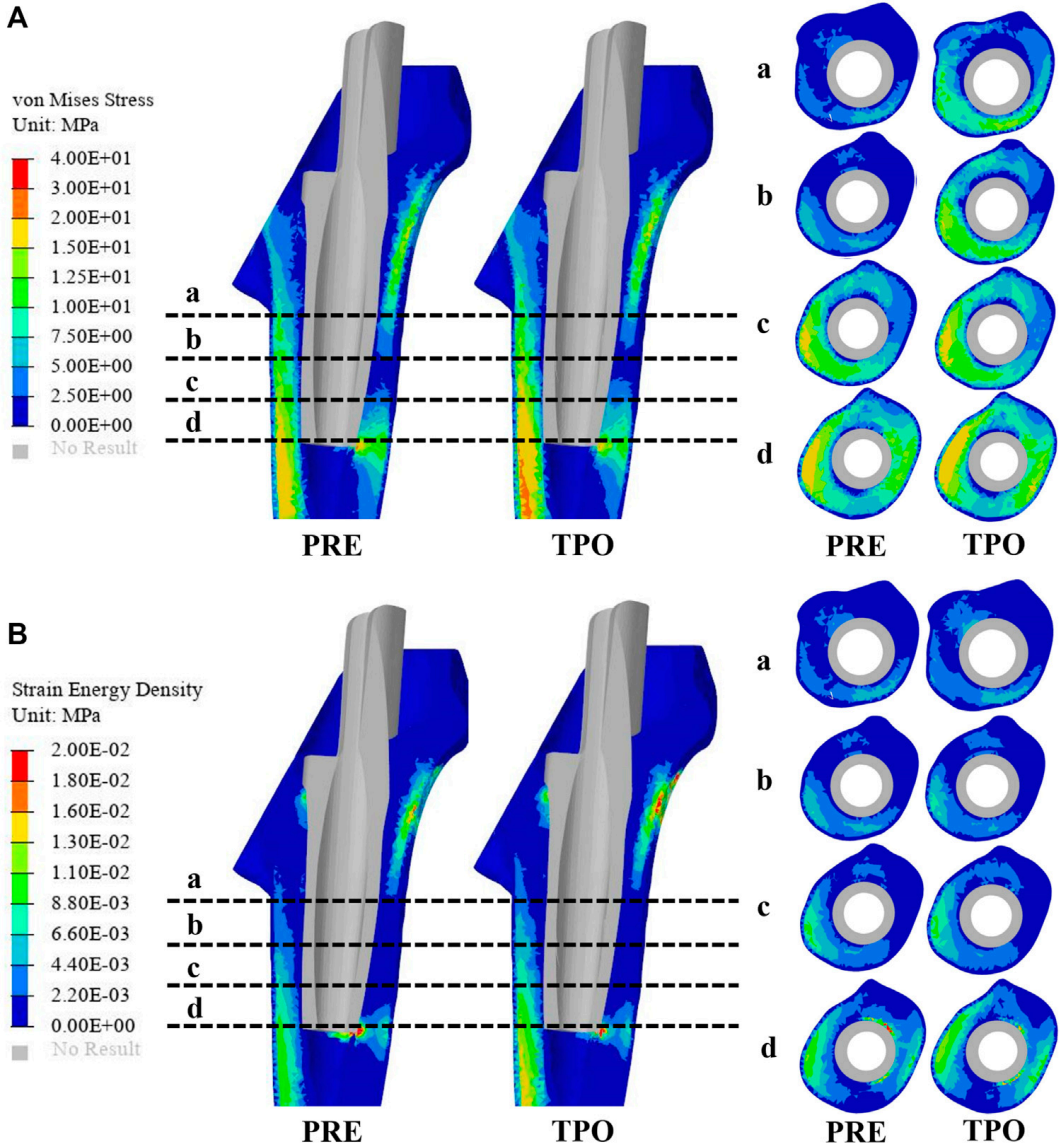
Comparison of the stress and strain energy density distribution on the femur between the original group (PRE) and optimized group (TPO) **(A)** Distribution of von Mises stress **(B)** Distribution of strain energy density. The a, b, c, and d indicate cross-section layers of femur.

In this study, quasi-static loading non-linear analysis was employed in the simulation procedure with 30 steps iterated until convergence, and the iterative method was performed using the Newton–Raphson approach. To evaluate the adjusted biomechanical effect between the original and optimization groups, the strain energy density (SED), maximum stress, stress distribution, and relative micromotion were selected using the Optistuct software program (Altair Engineering, Troy, MI, United States ). As a result, the differences in SED and the stress distribution between the original and optimized prostheses indicated an improved situation of bone resorption and stress shielding. According to the method of Zhang et al. and Wang et al., the average von Mises stresses were acquired at the medial and lateral femurs in 4 layers, which were located every 2 cm from the inferior border of the lesser trochanter to the bottom of the sleeve prosthesis to explore stress shielding (Figure 3) (Wang et al., 2018; Zhang et al., 2020). At each iteration, failure analyses were performed using the maximum principal stress to guarantee the implant’s necessary strength level under daily walking gait conditions. In addition, the distribution of relative micromotion, which was defined as an index to discriminate bone ingrowth requirements, was obtained from the relative distance of the opened gaps between the nodes at the contact surface. Statistical analyses were performed using the SPSS version 26.0 software program (IBM Corporation, Armonk, NY, United States ). The significance level was *p* < 0.05.

## Results

### TO Results

The density distribution of the sleeve prosthesis was mainly distributed on the top medial side (20%) and bottom lateral side (20–40%), as shown in Figure 2. The top and bottom protective devices were redesigned based on the initial optimization. As a result, the volume of the optimized prosthesis when redesigned was reduced from 14786.56 to 10,129.13 mm^3^, achieving a 31.5% weight reduction.

### Stress

**FIGURE 4:**
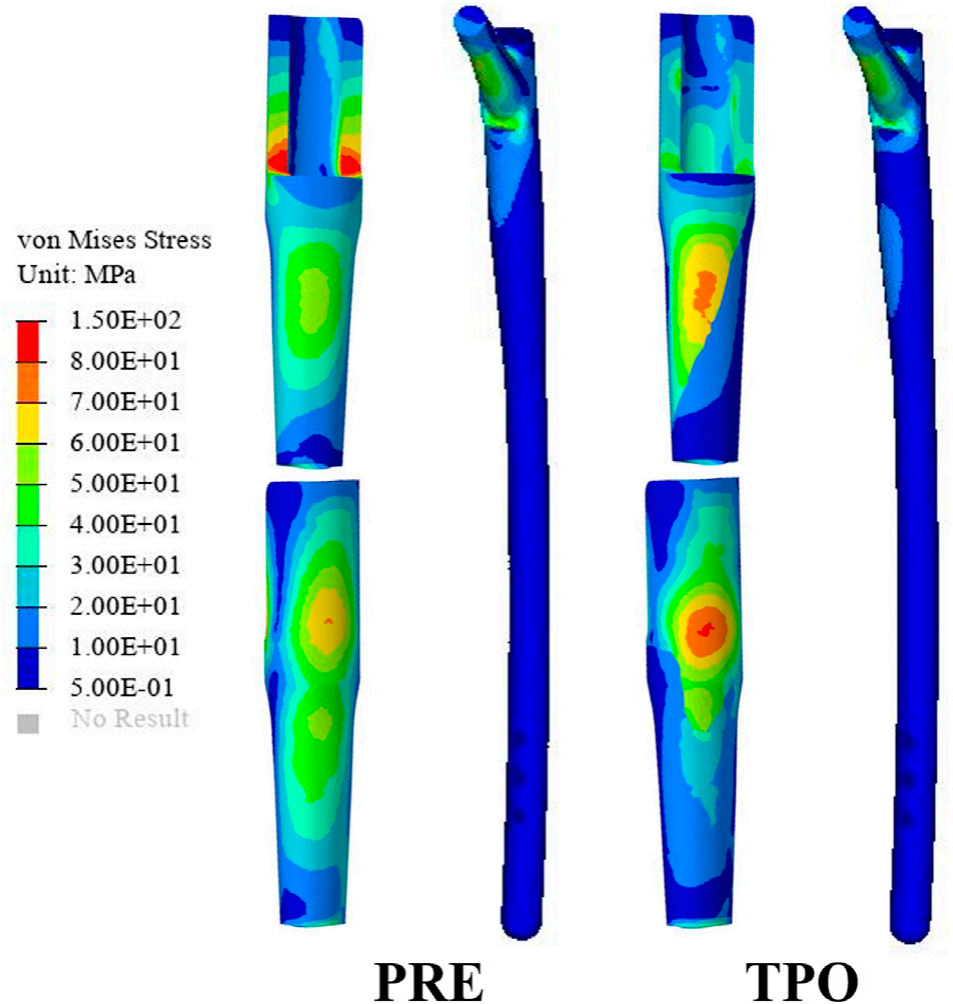
Comparison of the stress distribution on the prosthesis between the original group (PRE) and the optimized group (TPO).

**FIGURE 5:**
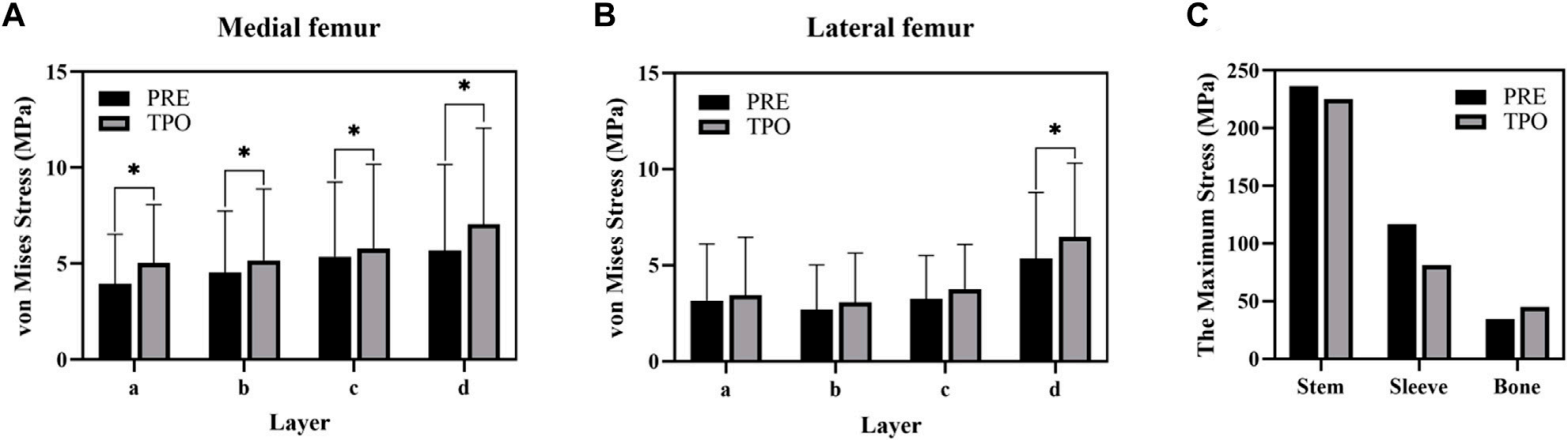
Comparison of the stress result **(A)** The average stress in the medial femur **(B)** The average stress **(C)** The maximum stress in components. The * indicates *p* < 0.05.

As shown in Figure 3A, the stress distribution in the proximal femur was observed to increase in most regions after optimization. Compared to the original group the average von Mises stress of all selected femoral layers was significantly increased on all medial sides (a–d) and the bottom lateral side (d) (*p* < 0.05) in the optimized group. The stress distribution in the prosthesis showed that optimized regions had a smaller concentration area than that in the original group (Figure 4). In addition, the maximum stress had reductions of 30.33% (81.31 MPa) and 4.74% (225.2 MPa) in back part of sleeve and neck part of stem prosthesis and a 29.52% (44.88 MPa) increase in femur compared to the original groups (116.7, 236.4, and 34.65 MPa) (Figure 5).

### SED

The results of SED in the proximal femur of the original and optimized groups are shown in Figure 3B. The distribution of SED in the proximal femur showed that most regions of the optimized group were increased. The maximum SED in the original and optimized groups experienced an increase of 50.97% (from 0.086 to 0.175 MPa).

### Relative Micromotion

**FIGURE 6:**
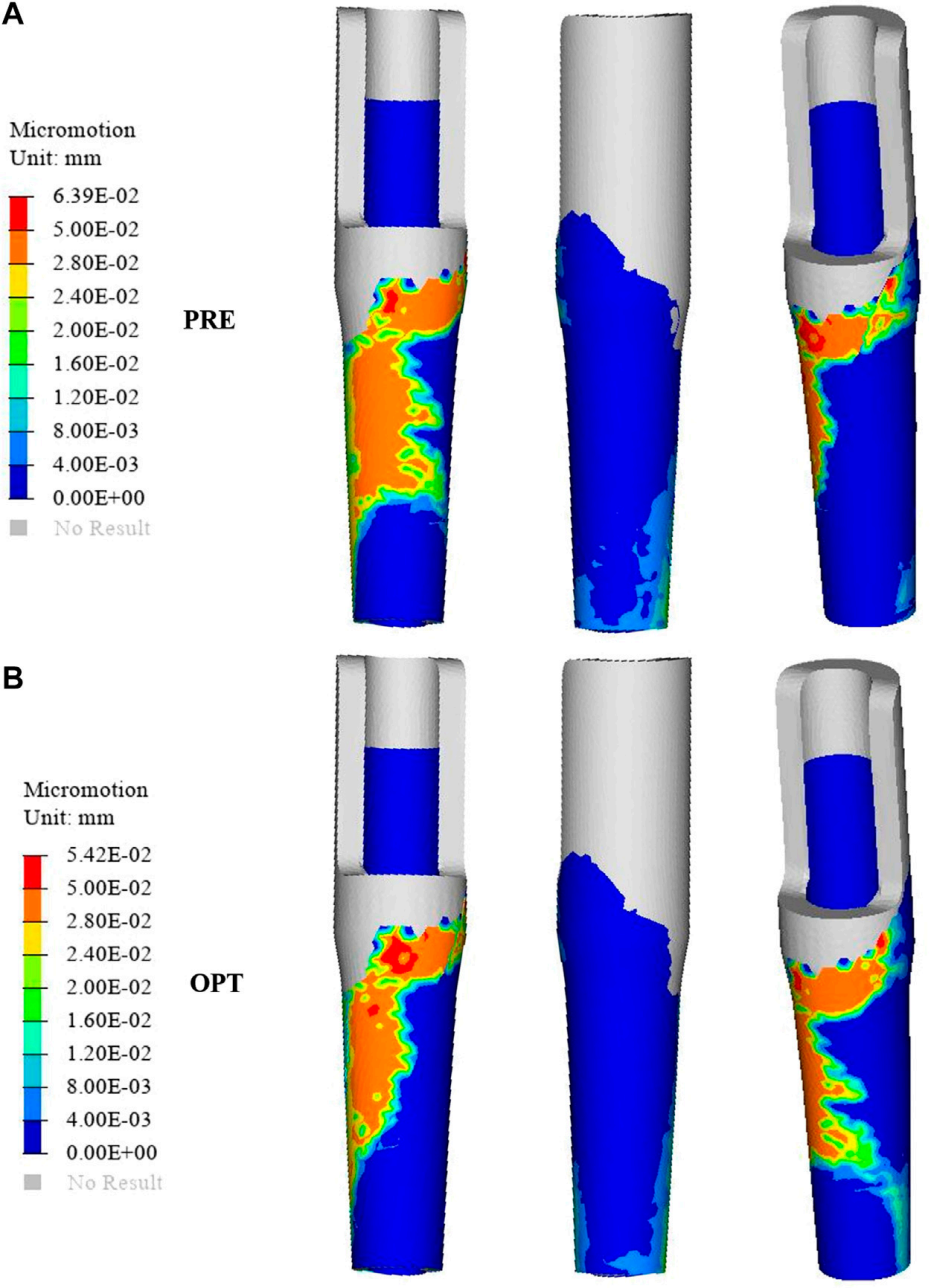
Comparison of the relative micromotion distribution between the original group **(A)** and the optimized group **(B)**.

The distribution of relative micromotion between the sleeve prosthesis’s external surface and the femur’s internal surface is presented in Figure 6. The regions with relative micromotion >28 μm in the optimized group showed a 24.5% increase (from 76.79 to 101.61 mm^2^) compared to the original group. In addition, the maximum relative micromotion decreased by 15.18% (from 63.9 to 54.2 μm) in the optimized group.

## Discussion

Successful bone defect surgery necessitates the implantation of both axially and rotationally stable components in the presence of physiological forces. While the choice of prosthesis has been controversial in previous studies, the use of extensively porous-coated stems in THA has, for many years, been the standard by which to address these defects (Wallace et al., 2020). Biomechanical challenges in such cases may include poor proximal bone stock, compound bone loss from a loose primary implant moving against the surrounding bone, and additional bone loss in initially installed prostheses (Wiik et al., 2021). The most severe problem caused by this alteration is stress shielding and wear failure, which would cause bone resorption and aseptic loosening around the prosthesis with long-term fixation (Wallace et al., 2020). A novel prosthesis design philosophy involving a porous metal material, surface treatments, and prosthesis geometry design was applied in this study. Although short-stem prostheses positively affect standard THA, remaining cortical bone in femur bone defect may make it challenging to provide sufficient prosthetic support and avoid additional bone collapse (Gotfried, 2007). In this study, in order to preserve the distal femoral bone reserve in the femoral cavity, obtain primary stability by biological fixation, and reduce stress shielding and referring to the successful application of porous short-stem prostheses in THA, a novel sleeve prosthesis was designed. Meanwhile, the point of the abductor’s muscle could be damaged during bone defect creation, leading to abductor weakness (Fuchs et al., 2021). A sleeve prosthesis could be applied to reconstruct abductor muscle attachment. Furthermore, to prevent the novel prosthesis from sinking, an intramedullary needle–type stem prosthesis that can be distally fixed with screws was added to the design. Then, a porous implant was optimized to improve its biomechanical performance based on the TO technique.

Porous structures are now widely used in orthopedic and dental bone implants. As a biomaterial, they provide pore interconnectivity and pore architecture for bone ingrowth for secondary long-term biologic fixation (Arabnejad et al., 2016). Bone ingrowth into an implanted structure is a highly complex phenomenon exhibiting biomechanical properties, including structural properties, strength, surface roughness, and biological conditions (Bartolomeu et al., 2019a). In addition, porous structures could regulate stress shielding to reduce bone resorption accordingly to match the stiffness of the local host bone. Some studies reported that the octet-truss structure is a good choice because of appropriate strength and bone ingrowth at high porosity (70% designed) (Feng et al., 2021; Korshunova et al., 2021). In addition, implants with different amounts of roughness have been proven to affect the ability of osseointegration via multiple factors, including enhancing the differentiation of osteoblasts, reducing the activity of osteoclasts, and promoting bone attachment to the implant surface and its mineralization. Bartolomeu et al., 2019a stated that 200 and 400 μm could be considered optimal pore sizes with suitable strength and roughness values to enhance bone ingrowth. Therefore, 200- and 400-μm octet-truss structures were chosen to be combined with the TO design. Then, an inhomogeneous femur model with muscle and joint force was simulated to realize an actual femur walking status in this study. The strength of optimized implants and their effects on host tissue were determined via FEA.

The stresses on the femur are used to indicate the stress shielding resulting from the various sleeve prosthesis insertions, as shown in Figure 3A. Compared to the bone applying original prosthesis, stress distribution was observed to increase in the femur model of the optimized group. The fully dense original prothesis exhibited a lower maximum principal stress (34.65 MPa) than the optimized group (44.88 MPa). This indicates that the load could be better transferred to the bone when altering the material properties from those of the porosity structure. This conclusion was also proved in our previous research (Liu et al., 2021; Zhao et al., 2021). Furthermore, the lesser trochanteric region is considered one of the most critical zones close to the calcar region of the femur and provides prosthetic stability (Wang et al., 2018). Because bone resorption occurs in this region, the implant design should involve some attention paid to minimizing stress shielding. The cross-section and integral view results show that this region experiences a greater stress increase, which is considered to yield a higher stress transfer to the bone to reduce stress shielding (Figure 3A, Figure 4). Mechanical stress is an essential factor in bone tissue remodeling, but an excessive load is one of the main inducers of fatigue damage (Maslov et al., 2021). According to the results in Figure 5, the maximum principal stress in the original group (bone, 34.65 MPa; prosthesis, 236.4 MPa) and optimized group (bone, 44.88 MPa; prosthesis, 225.2 MPa) were less than their corresponding fatigue strengths (cortical bone, 80–150 MPa; Ti_6_Al_4_V prosthesis, 310–610 MPa). This could indicate that the porous prosthesis’s strength is enough to sustain the loading requirement under walking conditions. The SED is another index used for evaluating stress shielding (Moussa et al., 2020). A higher SED result implies a lower stiffness, which could reduce the stiffness mismatch with bone (Wang et al., 2018). In our study, the maximum SED had a 50.97% increase in the femur of the optimized group (0.175 MPa) compared to the original group (0.086 MPa). Meanwhile, after optimization, the SED distribution increased noticeably in all femur layers, indicating decreased bone resorption and stress shielding (Figure 3B). Hence, the novel designed compound prosthesis would suit for some patients with bone defects and osteoporosis, because the poor femur proximal bone stock, stress shielding would be improved from the optimization design to deduce the risk of fracture.

Relative micromotion at the bone–implant interface is a vital consideration that may affect the biomechanical environment of bone ingrowth. Previous research has shown that micromotion >150 μm results in fibrous tissue formation, while adequate osseous contact and fixation can reduce micromotion and prevents fibrous ingrowth; meanwhile, micromotion of 30–150 μm results in bone and fibrous tissue formation, and micromotion of 28 μm results predominantly in bone formation (Pilliar et al., 1986). The success of long-term stability is dependent on the initial fixation of the implant, which is dependent on interface micromotion (Zhao et al., 2021). In this work, we designed a graded porous prosthesis to provide space for bone ingrowth and decreased the elastic modulus while increasing the friction coefficient to reduce relative micromotion. Here, the amount of micromotion was computed from the relative nodal sliding distance of mesh elements between the bone and the implant surfaces. As shown in Figure 6, the distribution of relative micromotion >28 μm decreased by 24.5% with the optimized prosthesis (76.79 mm^2^) compared to the original one (101.61 mm^2^). The affected region was mainly in the medial area of the prosthesis. Meanwhile, the maximum relative micromotion decreased by 15.18% (from 63.9 to 54.2 μm). The increase in micromotion can also be associated with postoperative pain; thus, this result indicated that the optimized prosthesis was capable of efficiently preventing micromotion, promoting bone ingrowth, and alleviating patients’ pain.

There are a few limitations to this study. First, characteristics specific to individual patients (bone density, dynamic biomechanical properties, and surrounding soft tissue) were not addressed. In addition, biomechanical changes relative to the original prosthesis were only analyzed by FEA. Biomechanical experiments and clinical applications should be considered to increase the integrity of future studies.

## Conclusion

In the study, a novel femoral sleeve prosthesis and intramedullary needle–type stem prosthesis were designed to treat proximal femur bone defects. Meanwhile, TO and a porous structure were applied to improve the prosthesis’ biomechanical performance to promote bone ingrowth and reduce stress shielding. Compared to the original prosthesis, the TO prosthesis exhibited reductions in weight (by 31.5%) and the maximum relative micromotion (by 15.18%) and an increased distribution of relative micromotion to promote bone formation (by 24.5%). In addition, the TO prosthesis could provide better stress distributions and reduce stress shielding. As a result, the novel designed compound prosthesis could serve as a model for clinical practice and a platform for future orthopedic surgery applications.

## Data Availability

The original contributions presented in the study are included in the article/Supplementary Material, further inquiries can be directed to the corresponding author.
